# Wave energy resource of Brazil: An analysis from 35 years of ERA-Interim reanalysis data

**DOI:** 10.1371/journal.pone.0183501

**Published:** 2017-08-17

**Authors:** Rafael Luz Espindola, Alex Maurício Araújo

**Affiliations:** 1 Rural Federal University of Semi-Arid–Campus Caraúbas, Caraúbas, Rio Grande do Norte, Brazil; 2 Department of Mechanical Engineering, Federal University of Pernambuco, Recife, Pernambuco, Brazil; Centro de Investigacion Cientifica y de Educacion Superior de Ensenada Division de Fisica Aplicada, MEXICO

## Abstract

This paper presents a characterization of the wave power resource and an analysis of the wave power output for three (AquaBuoy, Pelamis and Wave Dragon) different wave energy converters (WEC) over the Brazilian offshore. To do so it used a 35 years reanalysis database from the ERA-Interim project. Annual and seasonal statistical analyzes of significant height and energy period were performed, and the directional variability of the incident waves were evaluated. The wave power resource was characterized in terms of the statistical parameters of mean, maximum, 95^th^ percentile and standard deviation, and in terms of the temporal variability coefficients COV, SV e MV. From these analyses, the total annual wave power resource available over the Brazilian offshore was estimated in 89.97 GW, with largest mean wave power of 20.63 kW/m in the southernmost part of the study area. The analysis of the three WEC was based in the annual wave energy output and in the capacity factor. The higher capacity factor was 21.85% for Pelamis device at the southern region of the study area.

## Introduction

In recent decades, the world has been seeking to find environmentally sustainable energy solutions. For this, a great scientific effort in research on wind, solar, biomasses, wave and other renewable sources have been done. On a global scale, several wave power assessments have been done since the 1960s. It can be cited as examples the works [1,2].

Because of the large spatial dimension covered, studies done globally are only indicative of the potential of each region, requiring local analysis for better accuracy. In Europe, several studies have been conducted, for example, [3–7]. In the other continents, the number of researches are smaller. In Asia, the works [8,9] can be mentioned, in the Americas [10,11], in Oceania [12,13], in Africa [14] and in more than one continent [15,16].

Zhou *et al*. [17] classified the wave power assessments based on the data sources such as: traditional buoy data-based method, altimeter data-based method and wind data-based method. The last class include the works that use wave models such as WAM (WAve Model), WW3 (WaveWatch-III), SWAN (Simulated WAves Nearshore), etc. Nowadays, wave models are the most used method for wave power assessment because it allows a much more comprehensive spatial and temporal analysis than the other two methods and with lower costs. This work falls in this last class, since it uses the results from the ECMWF (European Centre for Medium-Range Weather Forecasts) ERA-Interim wave model data to evaluate the wave power resource of Brazilian offshore area.

The aim of this study it to characterize the main ocean wave parameters, estimating the wave power potential along the Brazilian offshore using 35 years of reanalysis data publicly available from the ERA-Interim project, produced by ECMWF. In addition, an analysis of the wave power output from three WECs is carried with the objective of evaluating the most suitable location for the production of this type of energy.

## Material and methods

### Study area

**Fig 1:**
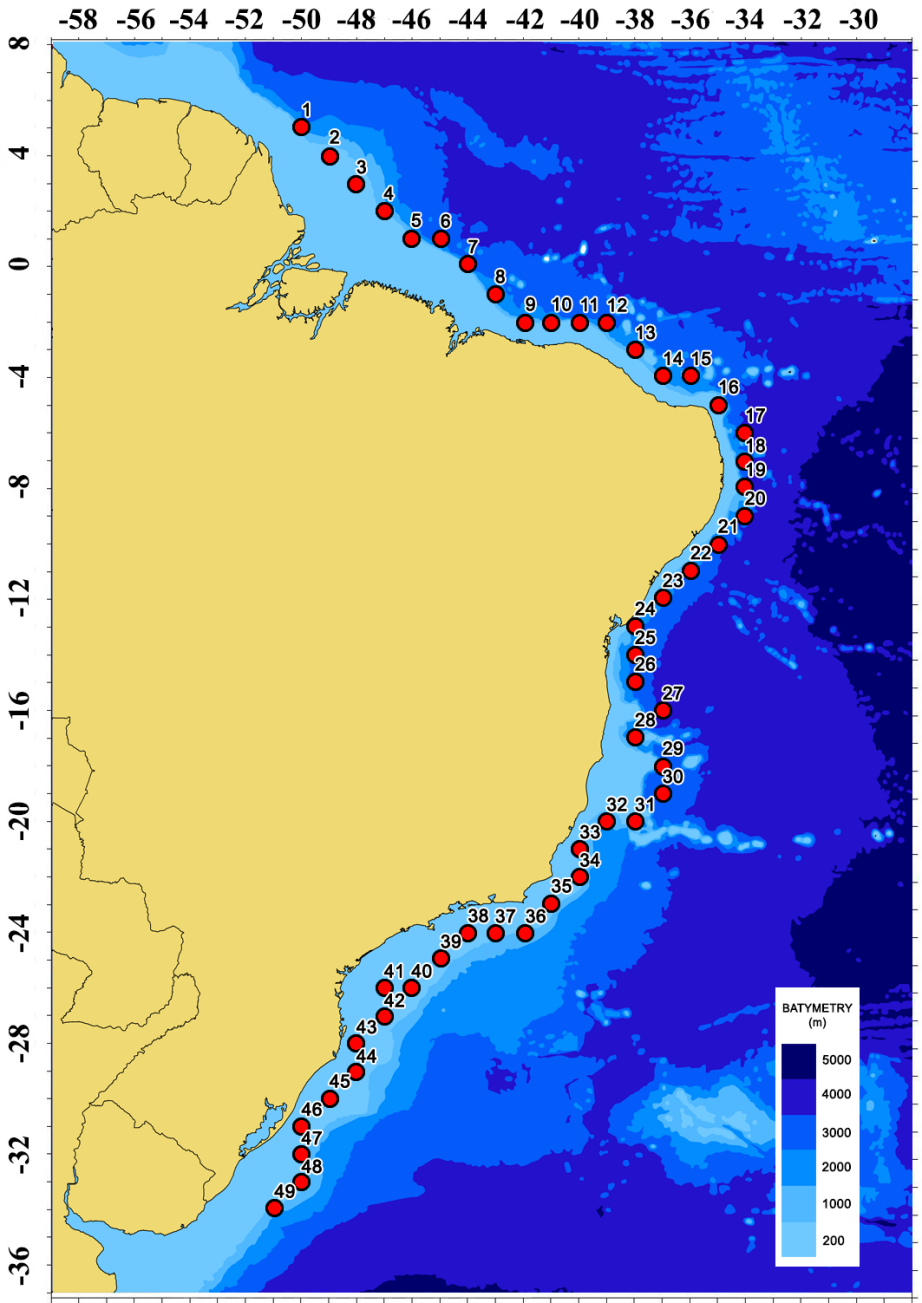
Bathymetry of the Brazilian Coast. Bathymetry source [18].

The study area is the Brazilian offshore region between latitudes 8°N-37°S and longitudes 28°W-59°W, with focus on the exclusive economic zone (EEZ). Located in the South Atlantic Ocean, Brazil has a long coast (more than 7000 km) and is composed of 26 States and a Federal District, of which 17 are located on the coastline. Fig 1 shows the study area with the bathymetry obtained from Natural Earth dataset [18]. Fig 1 also shows the location of 49 points spread over the Brazilian Coast used to characterize the wave climate.

Table 1 shows the location, depth and distance to the coast of the selected points. The distance from coast are approximated values.

**Table 1:** Locations, water depths and distances to the coast for the points considered.

Point	Latitude	Longitude	Depth (m)	Distance to coast (km)
P1	5°00’ N	50.00° W	-2316	151
P2	4.00° N	49.00° W	-486	206
P3	3.00° N	48.00° W	-365	247
P4	2.00° N	47.00° W	-1141	210
P5	1.00° N	46.00° W	-725	161
P6	1.00° N	45.00° W	-3551	187
P7	0.00°	44.00° W	-2296	149
P8	1.00° S	43.00° W	-2720	121
P9	2.00° S	42.00° W	-1901	60
P10	2.00° S	41.00° W	-1423	78
P11	2.00° S	40.00° W	-2263	76
P12	2.00° S	39.00° W	-3230	117
P13	3.00° S	38.00° W	-2506	88
P14	4.00° S	37.00° W	-2153	76
P15	4.00° S	36.00° W	-356	100
P16	5.00° S	35.00° W	-455	51
P17	6.00° S	34.00° W	-4310	122
P18	7.00° S	34.00° W	-3699	95
P19	8.00° S	34.00° W	-2277	96
P20	9.00° S	34.00° W	-3574	127
P21	10.00° S	35.00° W	-3431	80
P22	11.00° S	36.00° W	-2677	67
P23	12.00° S	37.00° W	-2728	65
P24	13.00° S	38.00° W	-2374	29
P25	14.00° S	38.00° W	-3286	104
P26	15.00° S	38.00° W	-3528	118
P27	16.00° S	37.00° W	-4032	220
P28	17.00° S	38.00° W	-2123	132
P29	18.00° S	37.00° W	-3582	252
P30	19.00° S	37.00° W	-3593	278
P31	20.00° S	38.00° W	-1467	207
P32	20.00° S	39.00° W	-1631	98
P33	21.00° S	40.00° W	-1320	60
P34	22.00° S	40.00° W	-288	114
P35	23.00° S	41.00° W	-116	81
P36	24.00° S	42.00° W	-1109	93
P37	24.00° S	43.00° W	-392	88
P38	24.00° S	44.00° W	-151	70
P39	25.00° S	45.00° W	-156	90
P40	26.00° S	46.00° W	-188	164
P41	26.00° S	47.00° W	-111	116
P42	27.00° S	47.00° W	-169	165
P43	28.00° S	48.00° W	-101	57
P44	29.00° S	48.00° W	-222	97
P45	30.00° S	49.00° W	-115	91
P46	31.00° S	50.00° W	-115	54
P47	32.00° S	50.00° W	-521	110
P48	33.00° S	50.00° W	-1012	168
P49	34.00° S	51.00° W	-1728	176

Historically, Brazil is a major user of renewable sources, with hydropower as the main source. However, despite all, few studies to harness wave power have been made. Among these few efforts one can mention Ricarte Beserra *et al*. [19], a pilot project to develop and install the first wave power plant of the Americas. Gonçalves *et al*. [20] presents as reasons for the lack of studies about the uses of wave energy in Brazil, the scarcity of wave data, the difficulty of attracting investors and the absence of institutional mechanisms that promote the development of such technology.

Among the few studies available, one can cite Estefen *et al*. [21] where the wave energy potential is discretized between latitudes 19°S and 32°S using 6.5 year of altimetry data from the satellite Topex/Poseidon, resulting in an annual mean wave power of 40 GW. Ricarte Beserra [22] used nearly two years of buoy data to measure the wave potential of one state in the Northeast Region of Brazil. As a result, the wave power resource evaluated monthly varies between 6 kW/m to 11 kW/m, with annual average of 7.7 kW/m. Contestabile *et al*. [23] assessed the offshore and nearshore (20 m isobath) wave power resource of a region in the south coast of Brazil. ERA-Interim reanalysis data were used to evaluate the offshore resource (15.25 kW/m) and the numerical coastal propagation model Mike21 SW was used for nearshore resource (11.43 kW/m). Pianca *et al*. [24] evaluated six points throughout the Brazilian Coast using the wave model NWW3 (NOAA Wave Watch III) with focus on significant wave height and wave period. The NWW3 was validated at one of the points from buoy data. The same mentioned buoy data was used in a previous work [25].

In addition, complete assessments of the wave power in Brazilian offshore were done by [26–28], with [26] and [28] using the 3^rd^ generation wave model WW3. Carvalho [26] is one of the most complete analysis about Brazilian Coast. In that, the Brazilian Coast is divided in ten regions and the monthly mean wave power is estimated. From the results, the more relevant area to install a wave energy converter (WEC) was the eastern part of the Northeast Region, with monthly mean wave power resource between 10 kW/m and 17 kW/m. Using the wave power results from [26], Fleming [29] estimated the Brazilian offshore resource is almost 91 GW. Souza [27] evaluate the wave power resource of offshore region to 160 GW. Moreover, Silva [28] carried a seasonal analysis of the wave power resource where it was observed that between the months of December and February the wave power are higher in the North and Northeast Regions of Brazil and lower in the Southeast and South Regions. During the period between June and August the inverse occurs.

All these works cited, except for [22,24,25], do not use direct measurements to estimate wave energy potential. This happens because the most part of the Brazilian coast is still unknown in terms of modern measurement techniques, and the few available measurements generally are spaced and targets petroleum activities. This lack of data can produce widely varying results such as between the studies [26] and [27], which yields a difference about 80% of the estimated potential.

### Reanalysis data

ERA-Interim is a global reanalysis produced by ECMWF. Its coverage period begins in 1979 and continues to this date. Products are available in a rectangular grid that includes a wide variety of surface parameters, describing the climate, the ocean waves and the land surface. They also include upper-air parameters, covering troposphere and lower stratosphere [30].

The wave model is an integral part of ERA-Interim. It is based on the 3^rd^ generation spectral wave model WAM two-way coupled to the atmospheric model [31] and includes a number of improvements in both physical and numeric aspects with respect to the previous reanalysis, the ERA-40. Among the presented improvements, the most significant for climate applications are the introduction of a solution to unresolved bathymetry effects and enhanced dissipation source term [30].

Altimeter wave height observations were assimilated into the wave model since the end of 1991 [30]. The validation was made against in situ observations of wave parameters obtained from buoys, platforms, and/or weather ships [30]. It is one of the most credible datasets according to Rusu and Onea [32], besides the various inherent errors present. It is the product of many years of outstanding scientific research and development, and was used directly or as input in many works [32–36] about wave energy around the world. Contestabile *et al*. [23], for example, uses the ERA-Interim data to estimate the wave power in a region of the Brazilian South Coast.

The ERA-Interim publicly available dataset used in this work has a spatial resolution of 1° x 1°, and a 6h time step (00h, 06h, 12h, 18h UTC). The parameters assessed were significant wave height of combined wind-waves and swell (H_s_), wave energy period (T_e_) and the mean three quantities were derived from the 2D-wave spectra as evolved by the wave model. Archived spectra were also obtained at a few specific locations.

### Methodology

The wave climate characterization was done for the 49 locations indicated in Fig 1, using almost 35 years of ERA-Interim reanalysis data. The study period was 01/01/1979–09/30/2015. In the characterization, the significant wave height, H_s_, and the wave energy period, T_e_, as obtained from the 2D wave spectrum, and were yearly and seasonally analyzed statistically. The seasonality was quarterly, with the periods: December, January and February (DJF); March, April, May (MAM); June, July, August (JJA); September, October, November (SON).

Using the ERA-Interim dataset for the whole study area, an overall view of the H_s_ and T_e_ was carried through the construction of maps for both parameters. For mapping, the GIS software SAGA^®^ was used. More information about the software can be obtained in the user’s manual (http://www.saga-gis.org). The function of the maps in this study is only qualitative.

The characterization of H_s_ was done in terms of statistical values of mean, maximum, 95^th^ percentile (95%) and standard deviation (*σ*). Additionally, the frequency of occurrence of wave above 2 m was calculated. T_e_ does not present a significant variation thus was characterized only in terms of mean. The directional distribution was also estimated and the bivariate distribution of occurrence in terms of H_s_ and T_e_. The directional distribution was discretized using bins of 10°.

From H_s_ and T_e_, the offshore wave power was estimated using Eq (1).

$$P=\frac{\rho g^{2}}{64\pi }T_{e}H_{s}^{2}$$ (1)

where *P* is the wave power resource (usually expressed in kW/m rather the W/m as Eq (1) suggests), *ρ* is the sea water density (kg/m^3^) and *g* is the gravity (m/s^2^). As well as for H_s_ and T_e_, yearly and seasonally statistical analysis was made for wave power, with the addition of a monthly statistical analysis. The same statistical parameters of mean, maximum, 95%, and *σ* were calculated. Additionally, three temporal variability analysis coefficients defined by Cornett [37] were estimated. They are the coefficient of variation (COV), the seasonal variability index (SV) and the monthly variability index (MV), respectively calculated by Eqs (2), (3) and (4).

$$COV=\frac{\sigma }{\mu }$$ (2)

where *σ* is the standard deviation of the wave power series, and μ is the mean wave power.

$$SV=\frac{P_{s1}-P_{s4}}{P_{year}}$$ (3)

where P_s1_ is the mean wave power for the highest energy season, P_s4_ is the mean wave power for the lowest energy season, and P_year_ is the annual mean wave power resource.

$$MV=\frac{P_{M1}-P_{M12}}{P_{year}}$$ (4)

where P_M1_ is the mean wave power for the highest energy month and P_M12_ is the mean wave power for the lowest energy month.

To evaluate the wave energy that can be converted, three WECs were selected. They are AquaBuoy [38], Pelamis [39] and Wave Dragon [40]. The amount of wave energy output that can be converted by each device are available in the power matrix provided by the developer. The power matrix consists of bins of significant wave height and wave period, where each cell informs the energy output to that specific sea state. Thus, Eq (5) can be used to calculate the annual electric power converted by a WEC (in kWh).

$$E=\sum_{i=1}^{n_{T}}\sum_{j=1}^{n_{H}}f_{ij}P_{ij}$$ (5)

where *f*_*ij*_ is the frequency of occurrence of the sea state corresponding to the *j*-th column and the *i*-th row of the power matrix, P_ij_ is the wave power output of the WEC for the sea state defined by the *j*-th column and the *i*-th row of the power matrix. References [41–43] uses an alternative method to calculate the power output of a wave farm.

As the maximum electric power output (rated power in kW) of each device are different, a coefficient of efficiency also was calculated. This coefficient is the capacity factor C_f_, that is defined by Eq (6) as the ratio between the mean wave power output (P_E_) and the rated power of the WEC (P_RATED_).

$$C_{f}=\frac{P_{E}}{P_{RATED}}$$ (6)

In addition, an analysis of electric power converted by the WEC as a function of the direction of the incident waves was performed. In this analysis, the WEC is considered without directional control and installed so that the optimum capture direction is aligned with the MWD at the site. The optimum capture direction depends on the devices. For attenuator devices (as Pelamis) the optimum direction was considered to be parallel to the main axis. For a terminator device (as Wave Dragon), the optimal direction was considered perpendicular to the main axis (for Wave Dragon, the main axis was considered parallel to the front of the ramp). Point absorber devices (as AquaBuoy), in general are direction independent, thus, only Pelamis and Wave Dragon were considered. In the analysis, the directional sector over which the incident waves were centered, was increased by 10° each time from 0° until it reached 360°, and, using the Eqs (5) and (6), P_E_ and C_f_ for each directional bin were calculated.

## Results and discussion

### Wave spectrum

The wave spectrum is the most important form in which ocean waves are described [44]. From the information presented by the spectrum, it is possible to determine its main parameters, such as significant wave height, wave period, wave direction and directional spread of the local wave climate. Thus, as pointed out in [45], the correct knowledge of the local wave spectrum is an important factor in the selection of the most appropriate WEC type for a location.

**Fig 2:**
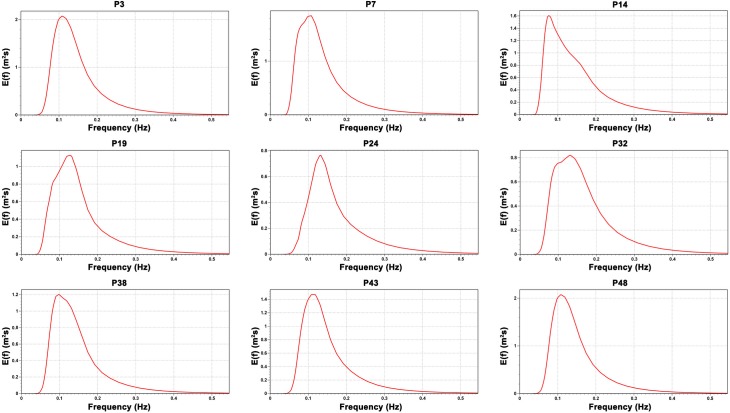
One-dimensional mean wave spectra for January. The figure shows the 1D monthly mean wave spectrum between 1979 and 2015 for 9 of the 49 studied points.

**Fig 3:**
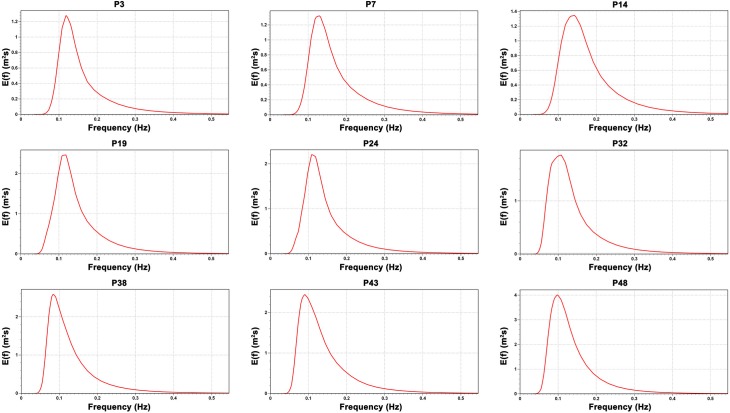
One-dimensional mean wave spectra for July. The figure shows the 1D monthly mean wave spectrum between 1979 and 2015 for 9 of the 49 studied points.

Fig 2 shows the one-dimensional (1D) mean wave frequency spectra for January between the years of 1979 and 2015 for 9 of the 49 selected points. Fig 3 shows the 1D wave spectra for July. The first three points (P3, P7 and P14) presents major amount of energy in January, while at the other six points (P19, P24, P32, P38, P43 and P48) the spectrum shows more energy in July. The pattern difference occurs because of the different weather systems that operate in the two regions. These weather system will be discussed later.

**Fig 4:**
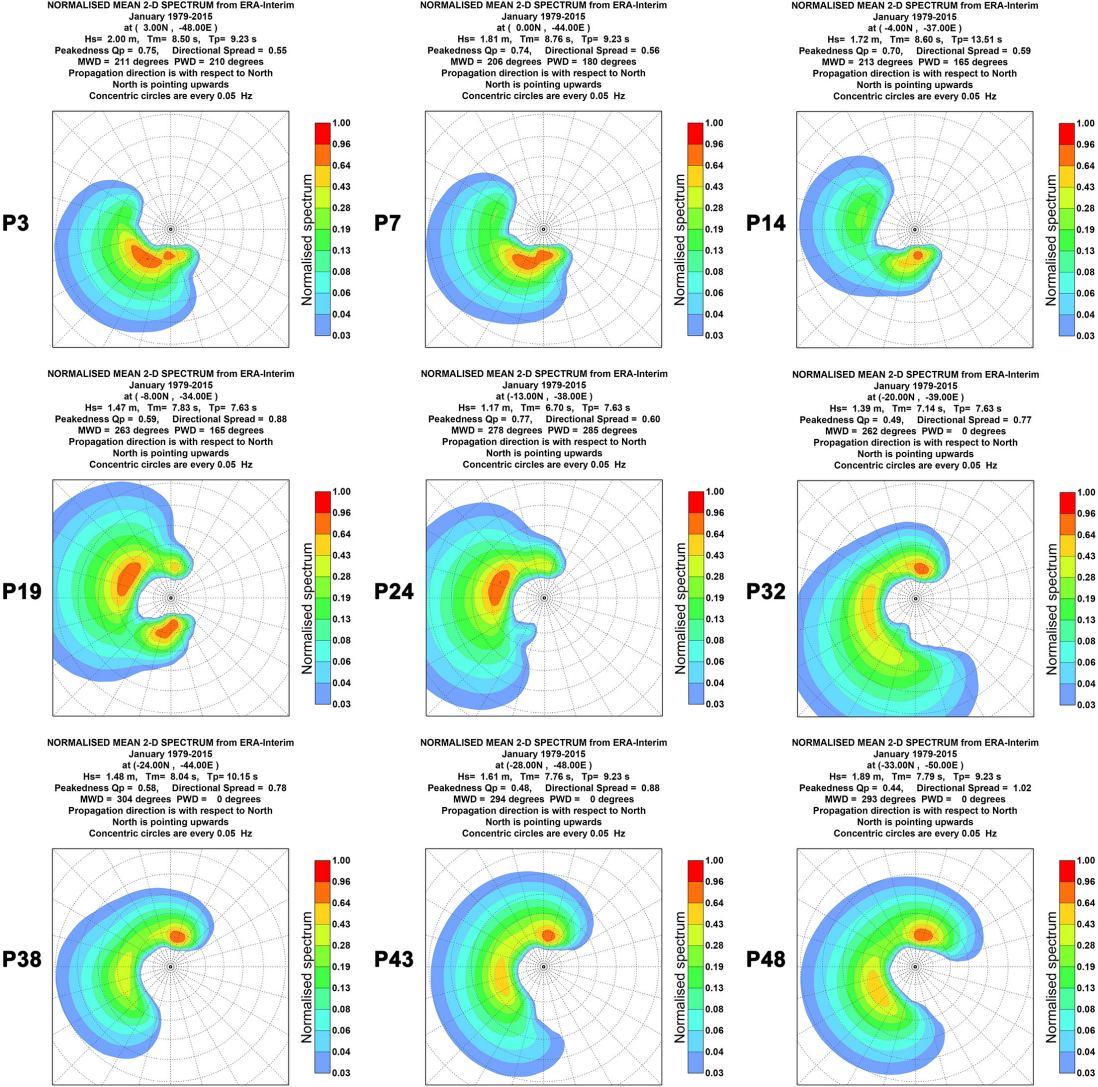
Bi-dimensional January mean wave spectra. The figure shows the 2D monthly mean wave spectrum between 1979 and 2015 for 9 of the 49 studied points.

**Fig 5:**
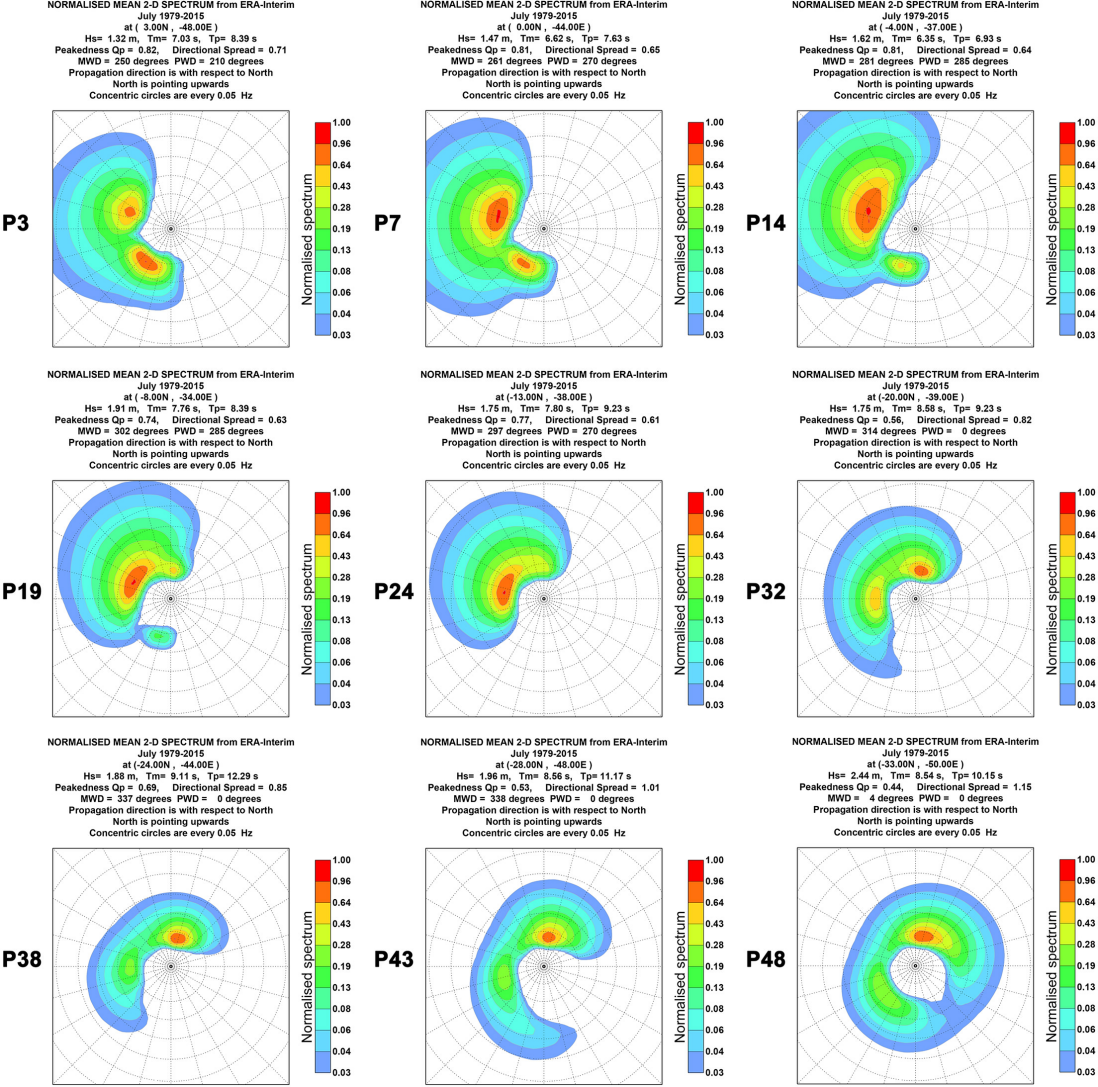
Bi-dimensional July mean wave spectra. The figure shows the 2D monthly mean wave spectrum between 1979 and 2015 for 9 of the 49 studied points.

Figs 4 and 5 shows the two-dimensional (2D) mean wave spectra for the same 9 points and for the same months used previously for the 1D wave spectra. The 2D wave spectrum describes the form in which the wave energy are distributed over frequency and direction. For almost all points shown in Figs 4 and 5, the 2D wave spectrum presents bi-modal characteristics with more than one wave systems being responsible for the wave energy. Besides the energy distribution shown by the wave spectra, Figs 4 and 5 present the values of significant wave height (H_s_), mean wave period (T_m_), peak wave period (T_p_), mean wave direction (MWD) and peak wave period (PWD) derived from the mean wave spectrum at each point. These parameters will be analyzed in more detail at the following sections.

Once the wave spectra present bimodal behavior, the MWD will be a mean value between the directions of two or more acting wave systems. Point P32 in Fig 5 shows clearly a system more energetic in the north direction and one less energetic in the west direction. In this case, the PWD was 0°, while MWD was 314°, a direction between the both systems where the wave energy is low. Thus, the use of the MWD alone can be a problem. For example, the commissioning of a WEC based only in the MWD may lead to a lower performance if the device is direction dependent. To avoid this kind of problem, a more detailed analysis of the 2D wave spectrum and the aid of other parameters, such as directional spread, are important to a complete further understanding of the local wave climate.

The directional spread is a measured of the dispersion around the MWD, similar to the standard deviation. Figs 4 and 5 show the normalized values for the 9 selected points. In Fig 4 it can be seen that an increase of this parameter occurred in January from 0.55 at point P3 to 1.02 at point P48. In July, as shown in Fig 5, occurred a decrease between point P3 (0.71) and point P24 (0.61) and another increase from point P24 to point P48 (1.15). In terms of harnessing the wave power, low values of directional spread are desired once the performance of some types of WEC are directional dependent.

### Analysis of the wave climate

**Fig 6:**
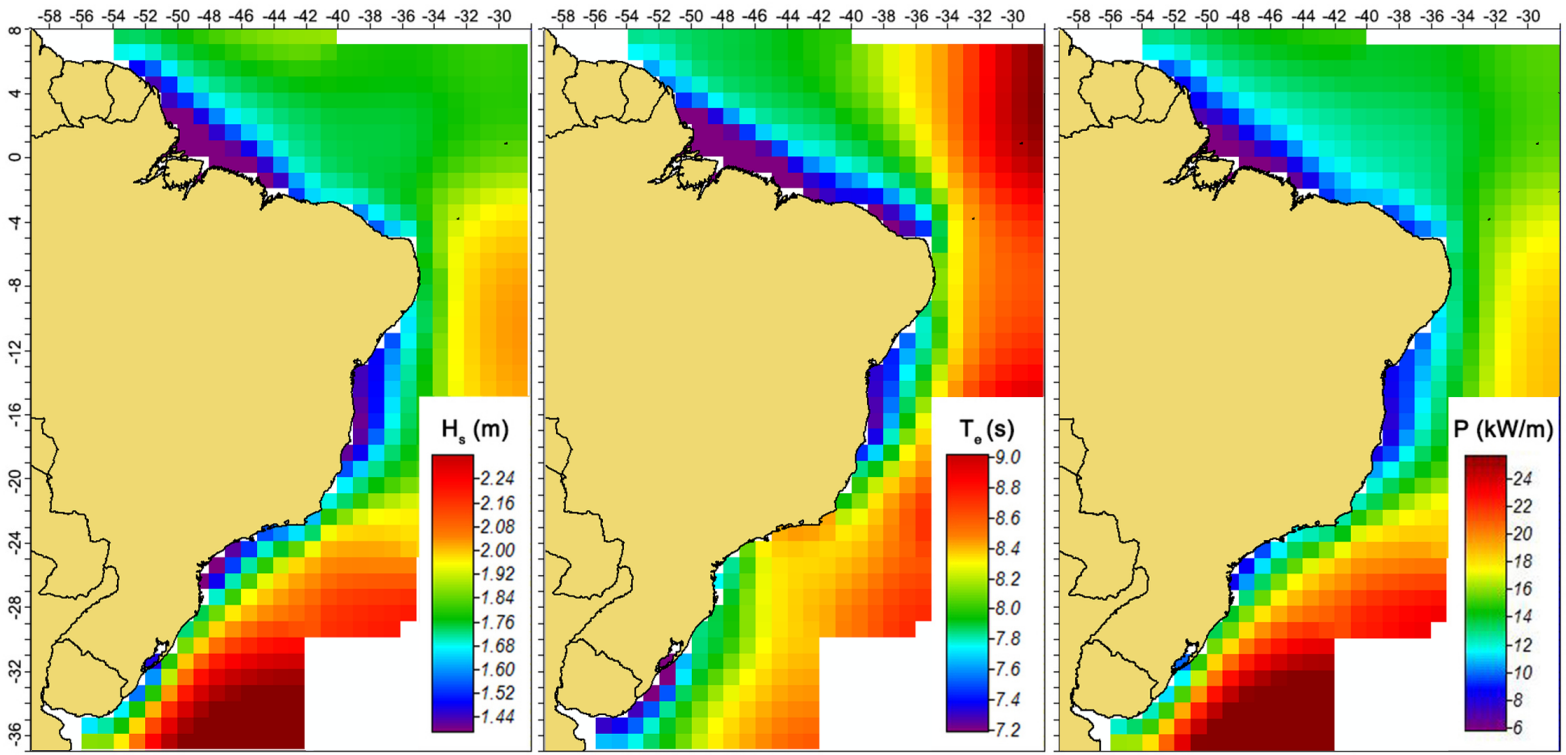
Mapping of the ERA-Interim data. (a) Annual mean significant wave height, (b) annual mean wave energy period and (c) annual mean wave power.

Fig 6 shows a map of annual mean H_s_, annual mean T_e_, and annual mean wave power resource on the study area, performed using the ERA-Interim data. The mapping was carried out using an inverse weighting distance interpolation. It can be observed an increase in average values related to the departure from the coast. This fact is mainly due to the increase in bathymetry. In the eastern area of the Northeast Region, the continental shelf is narrower than on the rest of the coast, as can be seen in Fig 1. Consequently, the annual mean H_s_ and the annual mean wave power exhibited significantly higher values than in the surrounding areas, where the continental shelf is wider. It is important to emphasize that Eq (1) was used also in points with bathymetry inferior at 100 m on the construction of the map showed in Fig 6, which introduce a certain error in some points. The same can be said about the interpolation used. However, as qualitative information the inaccuracy does not compromise the understanding of the behavior.

**Fig 7:**
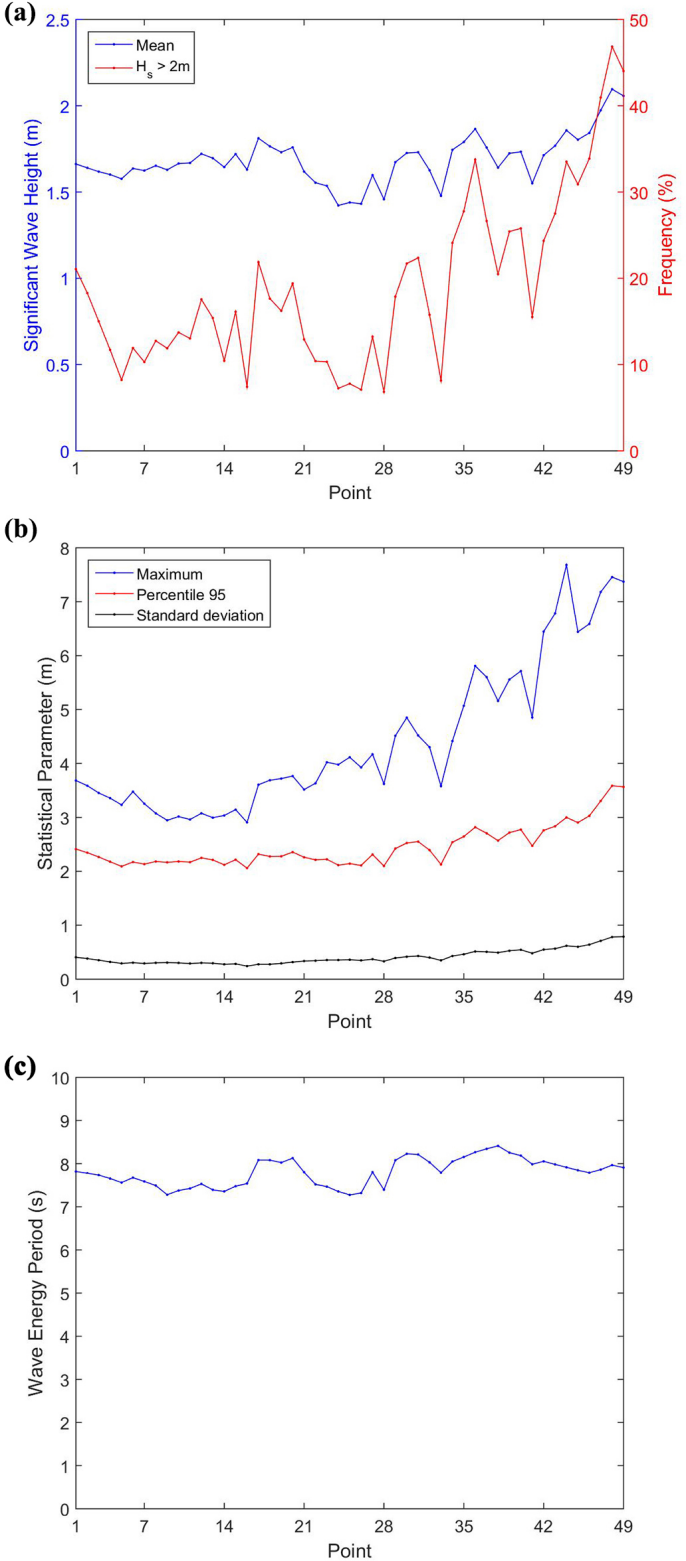
Parameters of the H_s_ and T_e_. (a) Annual mean H_s_ and the frequency of occurrence of H_s_ higher than 2 m; (b) yearly H_s_ statistical parameter of maximum, 95^th^ percentile and standard deviation; (c) annual mean T_e_.

From the 2D wave spectra, the wave conditions in the study area was characterized on the 49 points described in Table 1. Fig 7 shows the mean H_s_ and T_e_ values, the statistical parameters of H_s_, and the frequency of occurrence of H_s_ above 2m at each one of the 49 studied sites. Additionally, Fig 7B shows the yearly statistical analysis of H_s_. It can be observed that the largest significant wave heights occur in the southernmost of Brazilian coast, while the lower values are located in the central area, with the difference between largest and lowest H_s_ mean value of 0.68 m.

From the analysis of H_s_ mean values (Fig 7A), it can also be observed that the highest values occur in the southernmost area, between points P44 and P49, where the mean values are above 1.80 m, with the last two exceeding 2 m (P48 with 2.10 m and P49 with 2.06 m). The eastern (P17 to P20) and the southeast (P30 to P43) regions of Brazilian coast presents values in general above 1.70 m, with each highest mean values exceeding 1.80 m (P17 with 1.81 m and P36 with 1.87). Between points P1 and P16 and between points P21 and P29 occur the lowest values, with P24 showing the lowest result of all studied points, with 1.42 m. Unlike what happened with the significant wave height, the wave energy period did not show an increase trend to high latitudes, as can be seen in Fig 7C. Moreover, there isn’t any significant variation in mean values among the points, since the lower value is 7.28 s (P25) and the larger value is 8.41 s (P38), a difference of only 1.13 s.

According to Cavalcanti *et al*. [46] the trade winds and the Intertropical Convergence Zone (ITCZ) have an important role in the formation of waves in the region where P1 to P20 points are located. For the other points (P21 to P49) the South Atlantic Subtropical Anticyclone (SASA), the extratropical cyclones and the frontal systems are more relevant, mainly at the southern points. As the trade winds are nearly constant, there is little variation of H_s_ in the North and Northeast Regions, what explains the low range at points P1 to P20. The difference between the largest mean value (P17) and the lowest (P5) in this region is 0.21 m. On the other hand, the extratropical cyclones have large spatial and temporal variability, which cause a greater variation of H_s_ in the Southeast and South Regions.

The maximum H_s_ values also occur in the southern region with point P44 presenting the largest value of 7.68 m. However, unlike H_s_ mean values, the maximum H_s_ values present the lowest results only between P9 and P16, with 2.91 m in P16. Between P1 and P8, a decreasing trend can be observed, while between P17 and P46 an increasing trend occurs. An examination of 95^th^ percentile showed similar behavior to all other parameters analyzed with growing trend from northern to southern region, with largest value at P48 (3.59 m). Besides P48, the point P46, P47 and P49 present 95^th^ percentile value above 3 m. The little variation of this parameter, associated with the small standard deviation values, whose lowest and highest value are respectively 0.24 m (P16) and 0.79 m (P49), and closeness to the mean rather than to the maximum values, indicates a low variability of H_s_ on the analyzed points.

Regarding the percentage of waves with H_s_ greater than 2 m, the largest occurrence was obtained in the southernmost area between P47 and P49, with frequency greater than 40%, mainly in P48 where the frequency is 46.86%.

**Fig 8:**
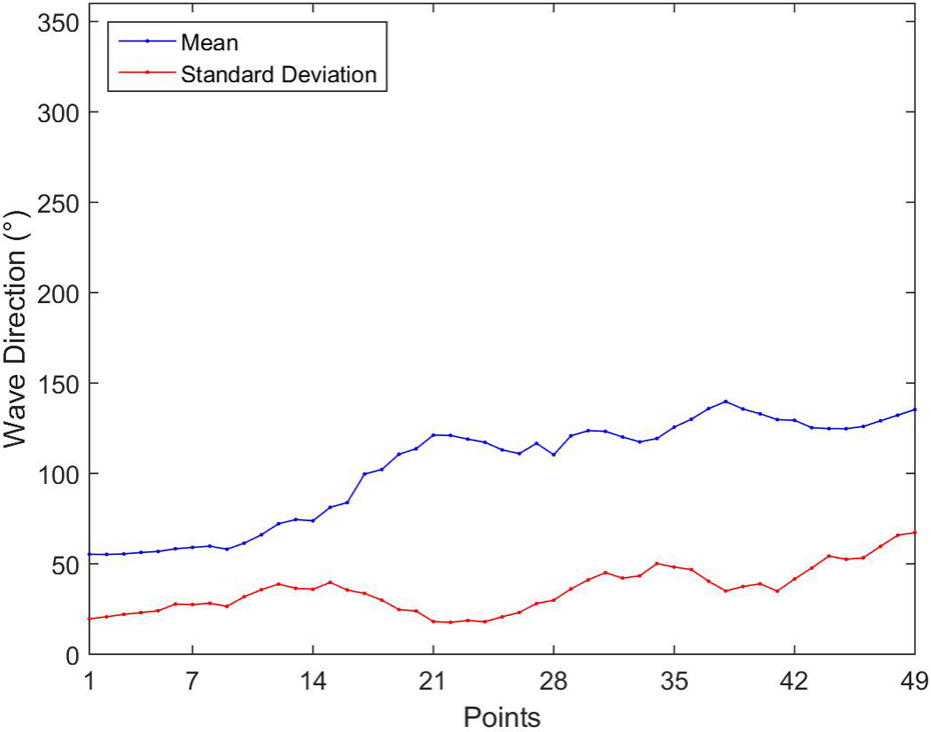
Parameters of the wave direction. Annual mean wave direction and standard deviation.

**Fig 9:**
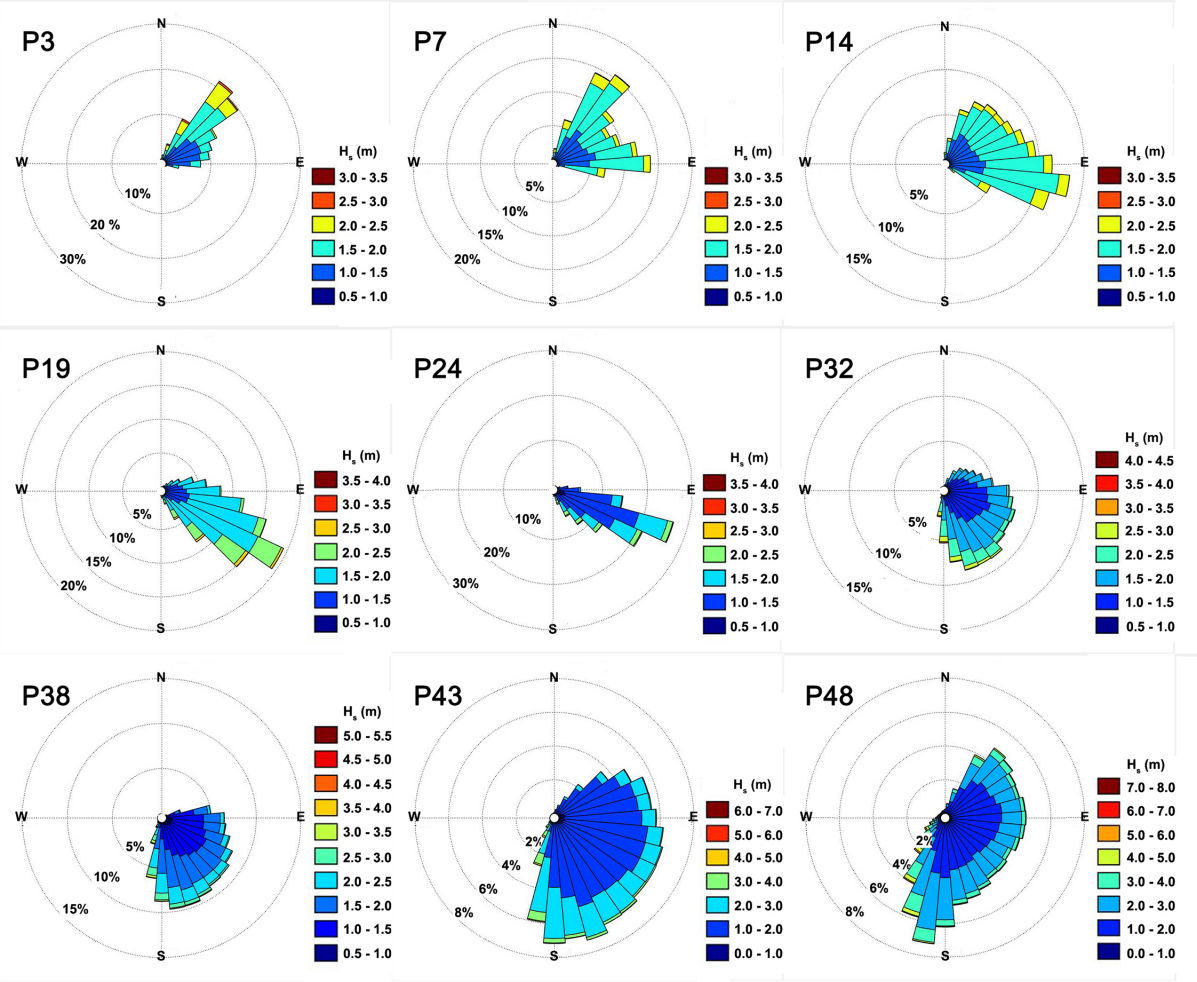
Directional distribution of significant wave height. The figure shows the frequency of occurrence of H_s_ per MWD for 9 of the 49 studied points.

Fig 8 shows the mean wave direction and the standard deviation of the studied points. Fig 9 presents the result of the directional distribution of H_s_ at 9 of the 49 points spread along the studied area. There was a clear distinction in mean wave direction between the first five points (P3, P7, P14, P19 and P24) and the other four points (P32, P38, P43, P48). The first five points showed a clear concentration of the MWD. At points P3 and P7, almost all MWD came from northeast direction. The most part of MWD at point P14 came from northeast, another part (approximately 20%) came from the east direction. At points P19 and P24, the MWD was mainly from southeast. At the other four points the waves had a large scattering, showing no predominant MWD. The difference between the first five and the last four points is caused by the aforementioned different meteorological systems that act in the regions. The behavior presented at these nine points can also be observed in the neighbors’ points.

**Fig 10:**
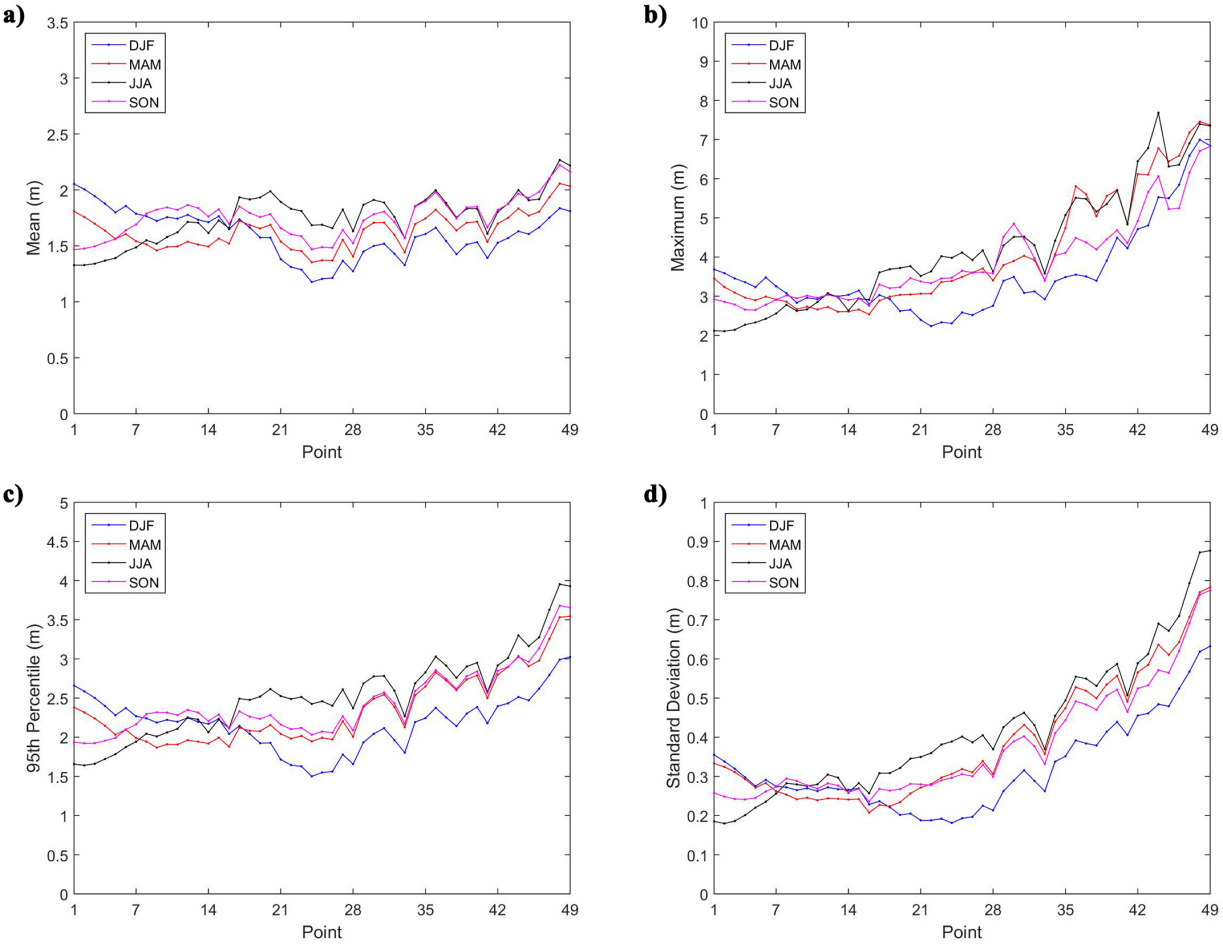
Statistical parameters of the seasonal H_s_. (a) Mean, (b) maximum, (c) 95^th^ percentile and (d) standard deviation of the seasonal H_s_ analysis at the 49 selected points. DJF: December, January, February; MAM: March, April, May; JJA: June, July, August; and SON: September, October, November.

A seasonal analysis was also performed in order to observe the temporal variability of the wave over the year. From the results shown in Fig 10 it was found that, there is an irregular seasonality of significant wave height among the 49 points. This was expected due to the large area covered by the study.

Fig 10A shows the H_s_ mean values for the four seasons. At the points P1 to P7, the largest H_s_ mean values happened at DJF period, while the smallest H_s_ mean values at JJA. The explanation for this is that during DJF the trade winds from the northern hemisphere are more intense than those from southern hemisphere, causing more intense waves in the Brazilian coast facing the northern hemisphere [47]. In addition, the positioning further south of the ITCZ in this period is another contributing factor [44]. On the other hand, during the JJA period the ITCZ is further north, thus points P1 to P7 are less influenced by systems that act in the Northern Hemisphere [28].

Points P8 to P16 had the largest mean value at SON and the smallest mean value at MAM. In turn, the other points (P17 to P49) had similar behavior with each other. For these points, the largest mean values occur mainly at JJA period, and the smallest mean values at DJF. In some points, as P41 and P46, the higher H_s_ mean value occur at SON. Despite the similar behavior, points P17 to P31 had a maximum mean value significantly higher than the second period of greater value, while for points P32 to P49 the two values are very close.

According to Cavalcanti *et al*. [46], the wave climate in southeastern coast of Brazil is greatly influenced by winds of SASA, passage of frontal systems and extratropical cyclones, as aforementioned. The last two factors occur less frequently in DJF period, which explains the low values shown in Fig 10. Similarly, the wave climate in the south coast it is also strongly influenced by extratropical cyclones, which justifies low H_s_ values at the same period. During the JJA period, these phenomena are more intense because of the winter in the southern hemisphere, which explains the largest mean H_s_ values occur at that period. The seasonal pattern of mean significant wave height is repeated for the maximum (Fig 10B), 95^th^ percentile (Fig 10C) and standard deviation (Fig 10D) of H_s_.

### Analysis of the wave energy resource

**Fig 11:**
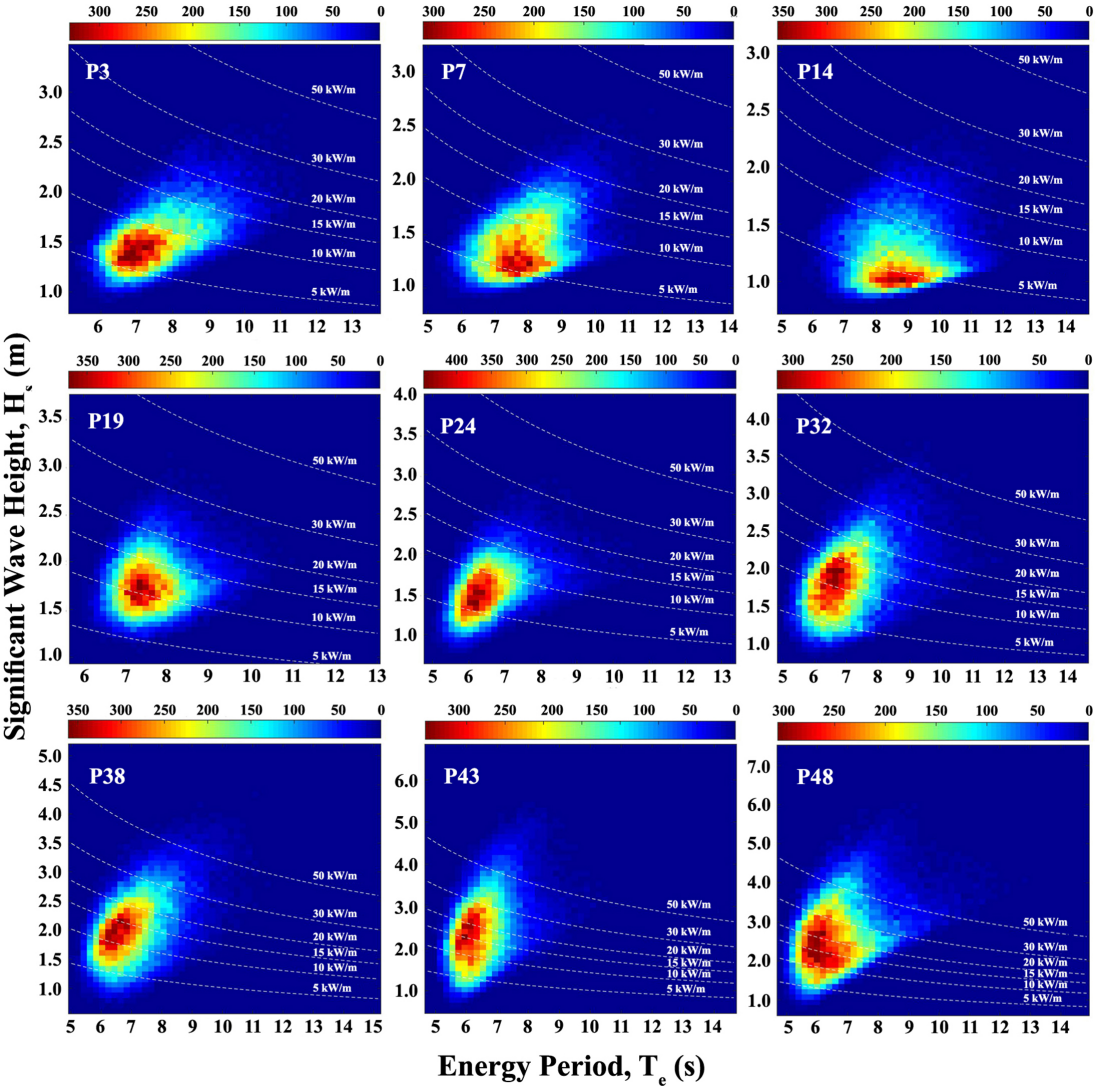
Bivariate distribution of occurrence in terms of H_s_ and T_e_. The figure shows 9 of the 49 points studied. The lines shows the pairs of H_s_ and T_e_ with the same amount of wave energy.

Fig 11 shows the yearly bivariate distribution of occurrence in terms of H_s_ and T_e_ that defines the sea state of a site at the same nine points used in the wave’s direction distribution. At the point P3 it can be observed that there is concentration of the occurrences in the range 1–1.5 m and 6–8 s. At points P7 and P14 the H_s_ range is the same, but the T_e_ range for P7 is 7–9 s and for P14 is 8–10 s. Point P19 presents more frequent values in the range 1.5–2 m and 7–8 s. Among these nine points selected, it is the point that presents the most concentrated results. At point P24, the H_s_ range with more occurrence is 1–2 m and the T_e_ range is 6–7 s. For the other four points (P32, P38, P43 and P48), the same T_e_ range of 6–7 s appears as the most frequent, but the H_s_ range slightly increase from P32 to P48. The H_s_ range is 1–2.5 m at P32 and P38, 1–3 m at P43 and 1–3.5 m at P48. In addition, it can be observed in Fig 11 the wave power resource isolines. Until point P24 the most frequent sea states presents a wave power resource of no more than 15 kW/m. After, in the points P32, P38, P43 and P48, it can be observed an increase in the most frequent wave power resources, reaching 30 kW/m.

**Fig 12:**
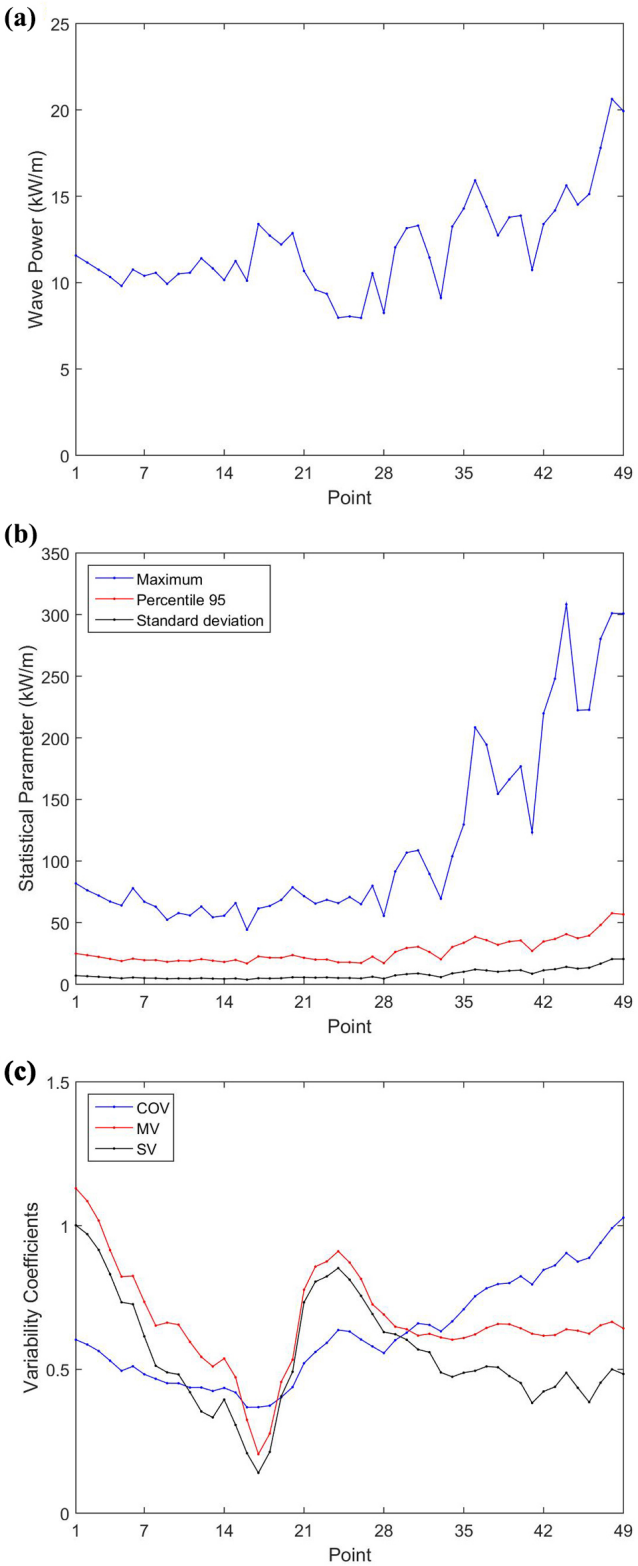
Parameters of wave power. (a) Annual mean wave power; (b) yearly wave power statistical parameter of maximum, 95^th^ percentile and standard deviation; (c) temporal variability coefficients of COV, SV and MV.

Fig 12 shows the annual average wave power and the statistical parameters (maximum, 95^th^ percentile and standard deviation) for the 49 selected points. The three coefficients of temporal variability proposed by Cornett [37] are also shown.

From Fig 12A it is observed that the region with the higher annual average wave power is the southern (P44 to P49) with the point P48 being the most energetic in the whole study area and presenting 20.63 kW/m. In the north and northeast regions (P1 to P29), point P17 stands out with 13.39 kW/m. Moreover, in the southeast region (P30 to P43), the point with largest annual average wave power resource is the P36 with 15.92 kW/m.

From the mean wave power resource an average value throughout the Brazilian Coast was estimated in 12.01 kW/m. Considering that the Brazilian Coast is 7491 km long [48], the total wave power resource available throughout Brazil can be calculated in 89.97 MW at an average distance of 127 km from the coast. This value of 89.97 GW is close to the value of 90.94 GW found by Carvalho [26] in a study carried throughout the whole Brazilian Coast too. To be considered viable, the distance of a wave farm from the coast need to be significantly lower. This will mean a reduction in the estimated wave power resource due to the energy losses that occur in intermediate and shallow water. Babarit *et al*. [49] mentions a loss of about 10% between deep and shallow water under typical wave conditions. However, for the implementation of a wave farm, direct measurements or modeling are necessary to take into account the dissipative effects of intermediate and shallow water.

The statistical analysis of the offshore wave power (Fig 12B) shows an increase of most of parameters with latitude, as happened with H_s_. The mean values of offshore annual wave power from points P1 and P49 was, respectively, 11.57 kW/m and 19.93 kW/m, and the annual maximum values was 81.84 kW/m and 300.83 kW/m. These values of annual average wave power are well below from what is used to consider a site as having a high resource. For comparison, Portugal has continental sites with up to 40 kW/m [50], more than two times the higher results found. However, the values calculated are close or better than that from other sites like Korean Peninsula [51] and Italy [52], each one with up to 11 kW/m. Moreover, the average annual values are in agreement with those obtained by previous studies for the region, such as [26,28].

Although the increasing trend from P1 to P49 for maximum values, 95^th^ percentile and standard deviation, this trend only intensified from point P29, with values between P1 and P28 presenting low variability. Thus, the low values of standard deviation found for points P1 to P28, combined with the low directional variability, indicates constant sea states in these points. In terms of power harnessing, this can compensate the low values of annual average wave power found.

**Fig 13:**
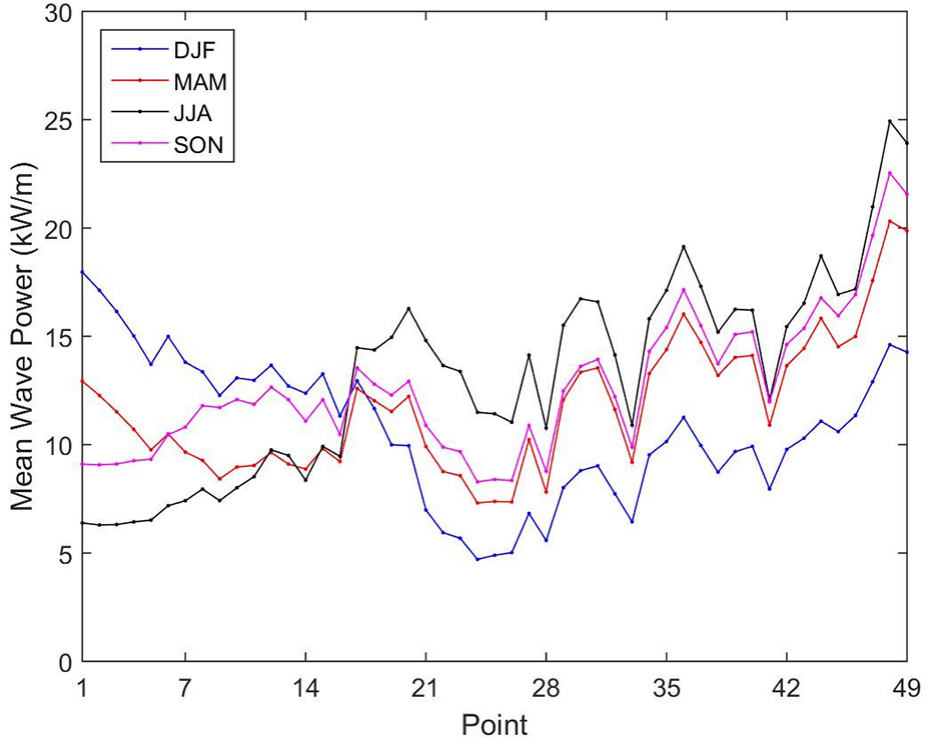
Average seasonal wave power resource. DJF: December, January, February; MAM: March, April, May; JJA: June, July, August; and SON: September, October, November).

Fig 13 shows that during DJF the points P1 to P16 are those with the largest wave power resource along the Brazilian Coast. This season is also the most energetic for these points throughout the year, with point P1 reaching 17.97 kW/m. According to Silva [28], this is explained by the low variability of the trade winds that operate in the formation of wave in the region during this period. In the following season, MAM, the mean wave power values from points P1 to P16 decreased while the values from points P17 to P49 increase until they reach maximum in JJA. During the JJA period, in points P1 to P16 is observed the smaller mean wave power values with the worst result being 6.29 kW/m at point P2. In turn, this season is the most energetic for points P17 to P49, with largest mean wave power values of 24.93 kW/m occurring at point P48. This pattern is mainly due to the winter in the southern hemisphere, a fact that also justified the increase in H_s_ and in T_e_ for the points at higher latitudes during JJA period.

**Fig 14:**
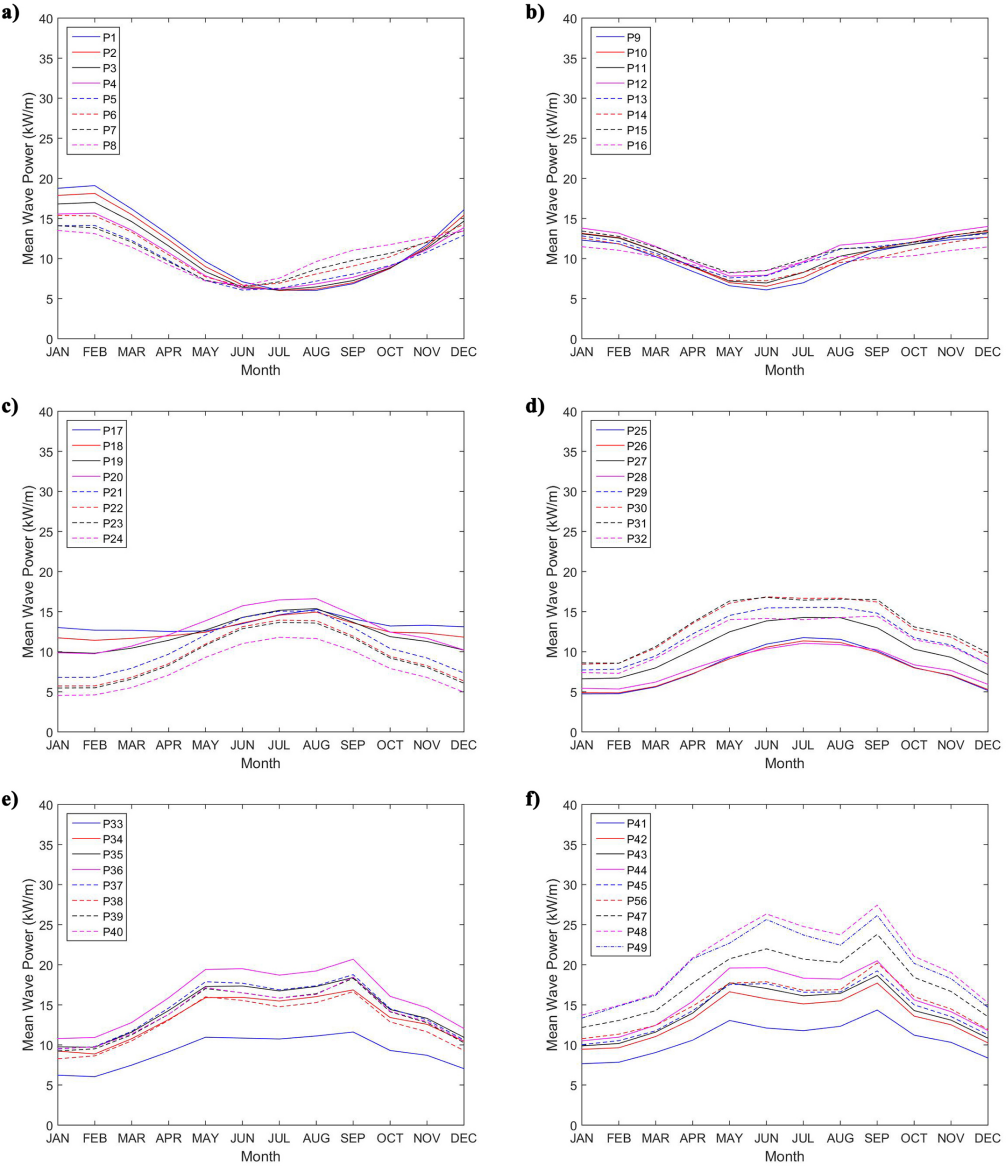
Monthly average values of wave power resource.

Additionally, Fig 14 shows the monthly average wave power resource over the 49 selected points. It can be observed that the points P1 to P16 had the largest mean wave power values occurring in the ending and beginning months of the year, and the lesser results in the middle of the year. For the other points (P17 to P49) the inverse occurs, and the most energetic mouths are those in the middle of the year. Besides the difference in temporal variability across the Brazilian coast, the range between the lower monthly mean wave power and the largest monthly wave power presents similar results to all points. The range of the point with the largest monthly wave power value (P48 with 27.44 kW/m in September) is 13.72 kW/m, which is significantly below than the ones found in some studies in different parts of the world. For example, Rusu and Onea [32] estimate in 726.2 kW/m the range in Iceland, more than 50 times that found at point P48. As points Portilla *et al*. [34], the low seasonal variability can be an important factor in the economic advantage of a wave power-harvesting project.

A temporal variability analysis was carried out. Sierra *et al*. [15] carried a similar analysis to evaluate the temporal variability of the wave power resource at the Atlantic coast of Morocco, and pointed that COV close to zero indicate low variability, between 0.85 and 0.90 indicate resource moderately unsteady, and COV greater than 1.2 denote considerable variability. For SV and MV, large values means larger seasonal variability, and values lower than 1 indicate moderate seasonal variability. Another study that used this temporal variability parameter is Sierra *et al*. [53], which study area was the island Lanzarote (Canary Island, Spain). For the Brazilian Coast, as can be seen in Fig 12C, the eastern region (P16 to P19) presents the lower temporal variability, with COV, SV and MV of 0.37, 0.14 and 0.21, respectively, in point P17. On the rest of the coast, COV increase in south and north direction. From north to south SV and MV starts with the maximum values of 1,00 and 1.13 in northern region (point P1), decrease until the lowest values on P17, as mentioned before, increase until P24 with SV equal to 0.85 and MV equal to 0.90, and decrease again to stabilize at SV and MV approximately 0.40 and 0.60. The low temporal variability in southern (P44 to P49), southeastern (P30 to P43) and eastern (P16 to P19) regions indicates good potential for WEC deployment in these areas, even with only moderate wave potential.

### Analysis of WEC performances

To evaluate the amount of wave energy output that can be delivered, three WECs was selected: Pelamis, WaveDragon and AquaBuoy. Each one of these WECs have different principles of conversion. The Pelamis is an attenuator device, the WaveDragon is an overtopping (and terminator) device, and the AquaBuoy is a point absorber device. The energy output of a WEC is indicated by power matrixes, where each bin of energy, defined by intervals of significant wave and wave energy period [54], has his own specific power output value. As pointed by Rusu and Onea [32], the power output from a WEC depends of a large number of factors, some unknown or imperfectly known. Because of that, even power matrices built from WECs widely experimentally tested, presents uncertainties in the power output. For Pelamis, Mackay *et al*. [55] found uncertainties of 20% in relation to estimated results. The power matrixes of Pelamis, WaveDragon and AquaBuoy were obtained from Rusu and Guedes Soares [54], and the main characteristics of each WEC are shown in Table 2. The recommended depth at each WEC was obtained from Rusu and Onea [32].

**Table 2:** Characteristics of the WECs selected.

WEC	Working principle	Rated Power (kW)	Recommended depth (m)
AquaBuoy	Point absorber	250	> 50
Pelamis	Attenuator	750	> 50
Wave Dragon	Overtopping	7000	> 30

**Fig 15:**
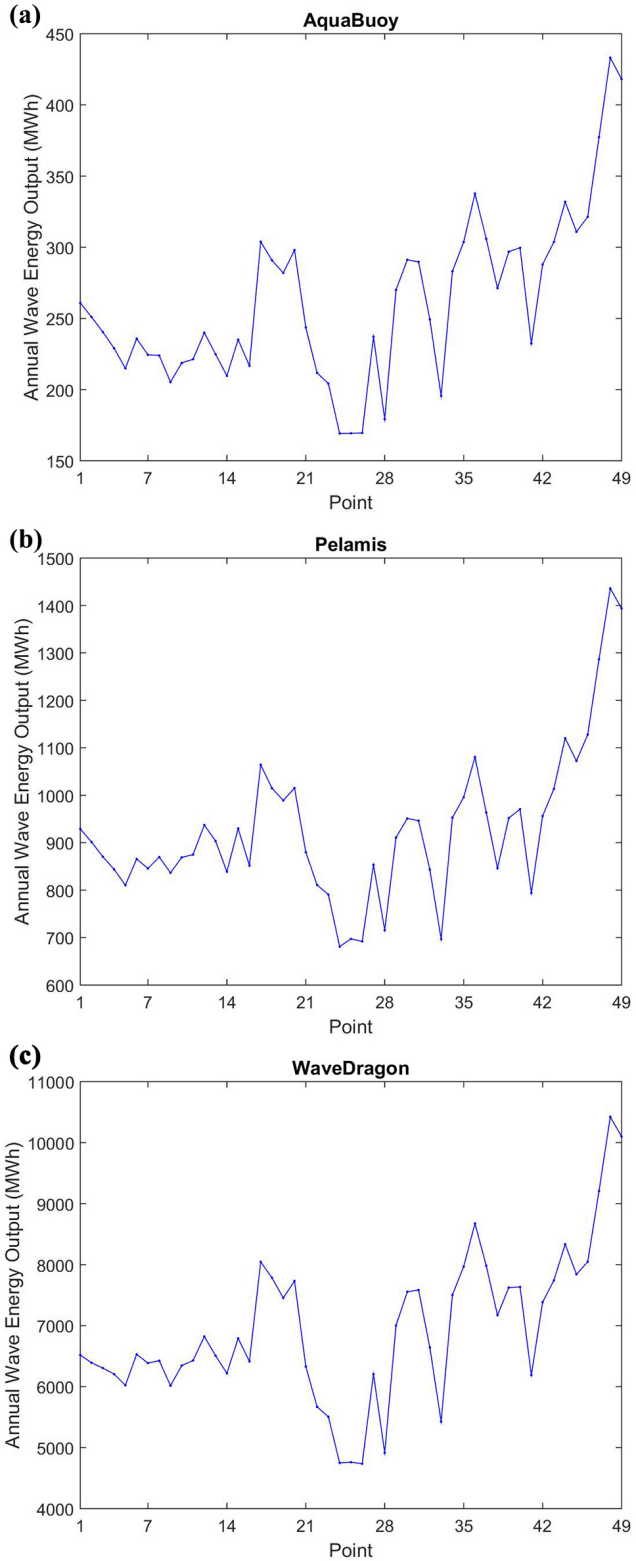
Annual wave energy output for analyzed WECs.

Using bivariate distribution of occurrence in terms of H_s_ and T_e_ (as those shown in Fig 11) to each point, with significant wave height and wave energy period with same bins as used in WEC power matrixes, the WEC energy output can be computed using Eq (5). The results are presented in Fig 15, and can be observed that the same points with best results for wave power resource are that the ones with best annual energy output. For the devices analyzed, the point with maximum annual energy output is P48 with 0.43 GWh (AquaBuoy), 1.46 GWh (Pelamis) and 10.42 GWh (Wave Dragon). The next two points with the most energy deployed are the neighboring points P49 and P47, respectively. Out of the southern sector, the most suitable point in northern sector to AquaBuoy is the point P1, with 0.26 GWh, in eastern sector is the point P17 with output of 0.30 GWh, and in southeastern sector the point P36 with 0.34 GWh. For Pelamis the best locations in each different regions are the same, with 0.93 GWh in P1, 1.06 GWh in P17 and 1.08 GWh in P36. For Wave Dragon the points are the same too, with output of 6.52 GWh in P1, 8.05 GWh in P7 and 8.67 GWh in P36.

An important fact that could be noticed in Fig 15 is the similar behavior of the devices throughout the study area. This can be explained in part by the similar characteristics of the sea states along the Brazilian Coast. Furthermore, it can be inferred from the devices’ power matrix [54] that they are developed to best performance in different conditions from those in Brazil. The most common T_e_ in Brazil are between 6 s and 9 s (as can be observed in Fig 11), while the Wave Dragon's best performance are obtained in T_e_ between 10 s and 15 s. For the other two devices the T_e_ range of best performance occurs in lower periods, 8 s to 12 s for AquaBuoy, and 6.5 s to 12 s for Pelamis. This fact impacts directly in the capacity factor of the devices, defined as the ratio between the average electric power and the rated power.

**Fig 16:**
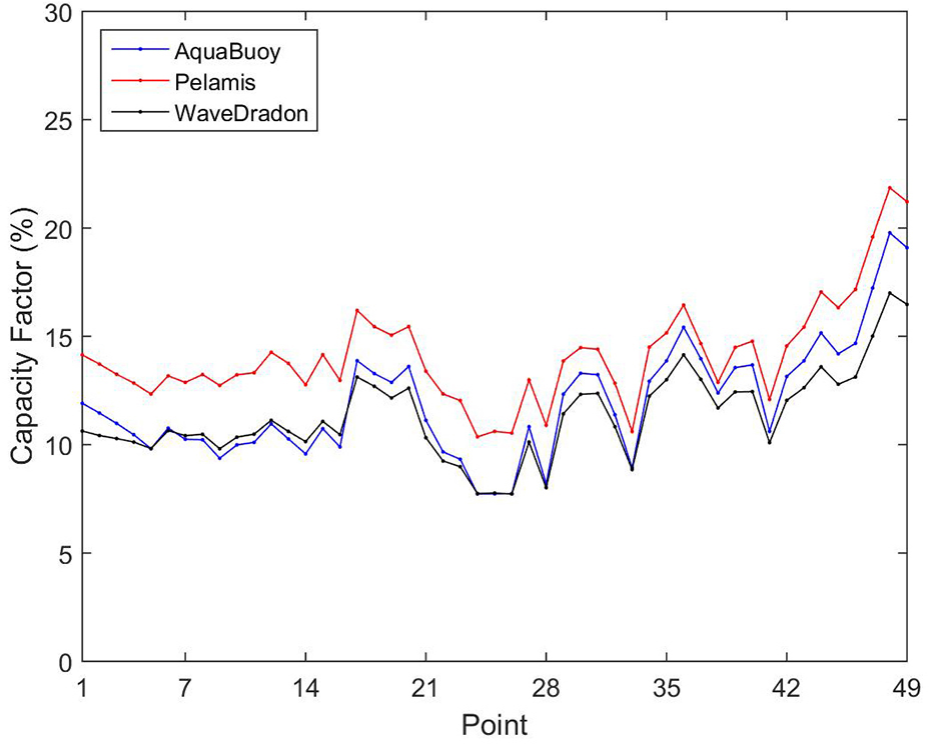
Comparison between the capacity factors for analyzed WECs. Aquabuoy (blue), Pelamis (red) and Wave Dragon (black).

Fig 16 presents the comparison of the capacity factor for the devices, and it can be seen that the three curves are similar with Pelamis standing out a little in relation to the others, mainly between P1 and P28. The points with best performance are the same as those with largest wave energy output in the study area. Pelamis reaches capacity factor between 10.36% and 21.85%, Aquabuoy between 7.73% and 19.78%, and Wave Dragon between 7.72% and 17.00%. These values are comparable to that found in another studies as presented in Table 3.

**Table 3:** Comparison between the capacity factor of points P1, P17, P36 and P48 in Brazil and some studies done in the North Atlantic Ocean and Mediterranean Sea.

Location	Reference	Mean wave power resource (kW/m)	Capacity Factor (%)		
			AquaBuoy	Pelamis	Wave Dragon
Brazil (P1)	-	11.57	11.91	14.14	10.63
Brazil (P17)	-	13.39	13.87	16.20	13.12
Brazil (P36)	-	15.92	15.42	16.44	14.15
Brazil (P48)	-	20.63	19.78	21.85	17.00
Alghero, Italy	[56]	10.30	8.70	9.40	8.80
Mazaro del Vallo, Italy	[56]	4.00	3.70	4.20	3.90
Madeira archipelago, Portugal	[32]	26.48	11.6	9.63	24.20
Canary Island, Spain	[32]	16.98	8.12	7.28	19.30
Iceland	[32]	45.05	17.90	15.30	33.40
Azores islands, Portugal	[32]	37.59	22.20	13.10	32.00
Morocco	[32]	29.94	-	14.76	25.51

Observing Table 3, the capacity factor in P48 are higher for AquaBuoy (19.78%) and Pelamis (21.85%) than that in Iceland (17.90% and 15.30%), even with the mean wave power resource in Iceland (45.05 kW/m) more than two times higher than in point P48 (20.63 kW/m). In fact, the result of Pelamis capacity factor at point P48 is the best of all locations presented in Table 3. For AquaBuoy similar results are found, with P48 presenting higher values than all, except in comparison with Azores islands (22.20%). For Wave Dragon the results for Brazil are inferior to those in locations at North Atlantic Ocean. This probably is consequence of the aforementioned power matrix of the device that presents best results in higher wave energy period bins. The results presented in Table 3 seem to indicate that the mean wave power resource is not the only determinant factor for the productivity of a wave farm. Similar conclusions were drawn by Contestabile *et al*. [23] and Babarit et al. [49]. As pointed by Contestabile *et al*. [23], the parameter wave power resource does not capture temporal and direction distribution of the incident wave and the limitations of WECs in capturing energy in high and low sea states conditions. Because of this, the knowledge of the directional spread of the wave energy is an important tool in the WEC technology selection, and for a more realistic assessment of the wave power output [45].

**Fig 17:**
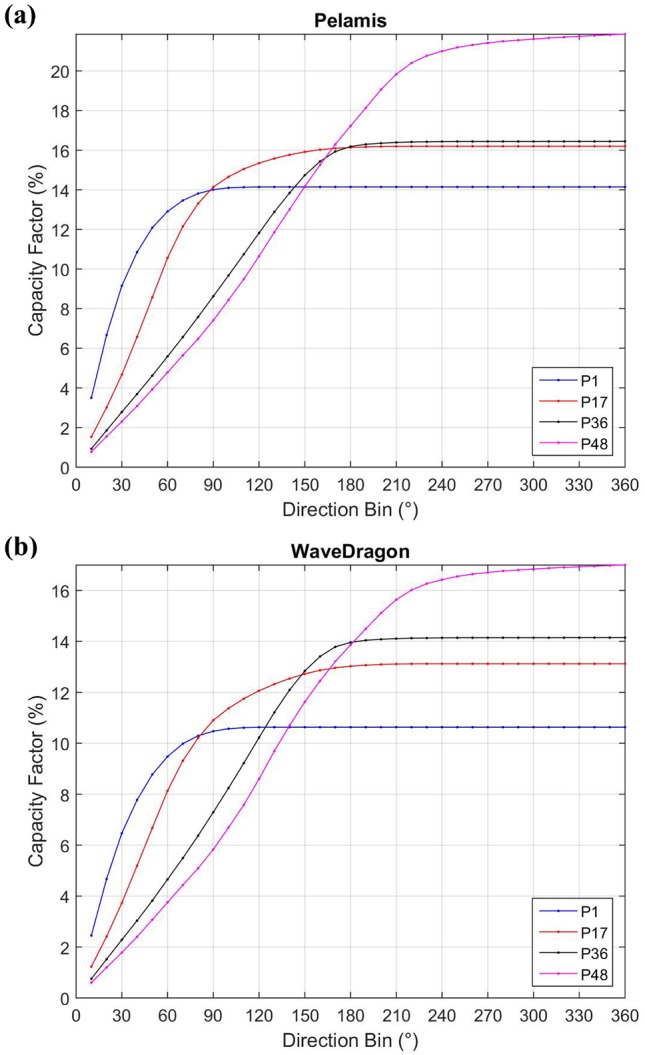
Variability of the WECs’ capacity factor in function of the directional distribution of the incident waves at points P1, P17, P36 and P48.

An analysis of the directional distribution influence on the WEC’s capacity factor was made. In this analysis, the devices are considered without any kind of directional control, and with the device direction of deployment equal to the mean wave direction calculated from the ERA-Interim database at the location evaluated. The directional sector over which the wave energy can be harvested are centered in the mean wave direction and are 10° increased each time until reach 360° As the AquaBuoy are a point absorber, and in theory independent of direction, the analysis was carried out only for Pelamis and Wave Dragon. The results are shown in Fig 17 for the points P1, P7, P36 and P48. It can be observed that the pattern are similar for both devices. Until a bin of 120° the capacity factor in P1 and P17 are higher than in points P36 and P48. This occur because of the major concentration of incident wave in few directions, as can be seen in Fig 9. As consequence, this 120° comprise almost all wave energy to these points. Thus, if the device does not have the capacity of capture incident waves from a large directional bin, locations with lower wave power resources (as points P1 and P17), but with lower direction distribution, a priori would present better capacity factors and would be more appropriate to be commissioned than sites with opposite conditions.

## Conclusions

From the wave characterization, it was found that the significant wave height and the wave power increases from north to south, reaching maximum values along the Southern Brazil coast. However, in the eastern part of the Northeast Region of Brazil the values are slightly higher than the adjacent areas. For the wave period, no significant pattern was observed.

The waves, in terms of direction, presents a clear distinction between those located in low latitudes and in high latitudes. At low latitudes, the incident waves at a given location present low variation in mean direction, concentrating on a narrow directional range. At high latitude the directional distribution is wider reaching values greater than 180°.

The Brazilian offshore presents an annual mean wave power resource between 7.97 and 20.63 kW/m. Compared with locations in the North Atlantic Ocean, where the potential can reach more than 40 kW/m, the values found in Brazil can be considered low to medium. However, they are similar, and even superior, to places like Korean Peninsula and Italy.

Although the values of the wave potential are only intermediate, the low temporal variability indicate that several Brazilian locations are suitable for the conversion of wave energy. These sites are not only concentrated in the southern area where the potential is highest.

In the southern region of the study area, the wave energy output at a location could reach up to 10.42 GWh if the Wave Dragon device is used. Despite presenting the highest production, Wave Dragon was not the devices with the highest values of capacity factor. The maximum capacity factor (21.85%) was obtained from Pelamis.

Despite being developed for the North Atlantic Ocean characteristics, the Pelamis and the AquaBuoy devices seem to adapt better to quieter sea conditions with little temporal variability, as occur in the study area. The Wave Dragon, on the other hand, outputs better in a more energetic sea with higher temporal variability.

Finally, the capacity factor, and consequently the production, is maximum for smaller directional bins in locations with low directional variability.
